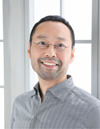# Message From the New Editor-in-Chief

**DOI:** 10.2188/jea.JE20210427

**Published:** 2022-01-05

**Authors:** Kota Katanoda

**Affiliations:** Division of Surveillance and Policy Evaluation, National Cancer Center Institute for Cancer Control, Tokyo, Japan

It is a great honor to address you as the new Editor-in-Chief (EIC) of the *Journal of Epidemiology*, the official journal of the Japan Epidemiological Association. I took over this honorable role from Dr Keitaro Matsuo, who devoted himself to the journal for 5 years. Since its launch in 1991, the *Journal of Epidemiology* has been a platform for scientific publication in the field of epidemiology and related social medicine. Originally, the journal mainly published articles on domestic epidemiological research and background healthcare systems in Japan. Under the initiative of former EICs, it expanded the scope to cover broader health and social issues in wider geographical regions. The number of submissions increased from approximately 150 in 2008 to over 600 in 2020, of which the submissions from overseas accounted for around 60%. More remarkable growth can be seen in the number of citations: the 5-year average annual number of citations surged by almost 15 times, from 180 in 2003–2007 to 2,800 in 2016–2020. Reflecting this, the impact factor of our journal has been steadily increasing, with the latest value of 3.21 in 2020. We have long been successful in being an open access journal, the status of which was endorsed by the DOAJ Seal awarded to our journal in 2015 by the Directory of Open Access Journals. Recently, our journal has dramatically improved the timeliness of our publication by adopting the early release of accepted version. Of course, these achievements are the result of dedicated work of all the authors, readers, and the former and current Editorial Board members.

In the era of COVID-19, scientific publication is faced with drastic changes in its role in society. We need to respond to ever-changing social conditions with unprecedented timeliness. The prevailing preprint style of publication is a pragmatic solution for this demand. However, there has also never been a time like today when scientific publications are under such pressure to produce credible information. The *Journal of Epidemiology* will continue to commit to the responsibility of providing our society with robust and long-lasting scientific evidence in a timely but steady manner.

Last but certainly not least, our journal always welcomes contributions from anyone in any form in the field of epidemiology and related social medicine.



Kota KATANODA, PhD
Editor-in-Chief
Journal of Epidemiology

Chief
Division of Surveillance and Policy Evaluation,
National Cancer Center Institute for Cancer Control, Japan